# Perspectives for 3D-Bioprinting in Modeling of Tumor Immune Evasion

**DOI:** 10.3390/cancers14133126

**Published:** 2022-06-26

**Authors:** Rafał Staros, Agata Michalak, Kinga Rusinek, Krzysztof Mucha, Zygmunt Pojda, Radosław Zagożdżon

**Affiliations:** 1 Department of Immunology, Transplantation and Internal Medicine, Medical University of Warsaw, 02-006 Warsaw, Poland; rafal.j.staros@gmail.com (R.S.); kmucha@wum.edu.pl (K.M.); 2 Department of Regenerative Medicine, Maria Sklodowska-Curie National Institute of Oncology, 02-781 Warsaw, Poland; agata.michalak2@pib-nio.pl (A.M.); kinga.rusinek@pib-nio.pl (K.R.); zygmunt.pojda@pib-nio.pl (Z.P.); 3 Department of Clinical Immunology, Medical University of Warsaw, 02-006 Warsaw, Poland

**Keywords:** tumor architecture, immune response, anticancer immunotherapies, in vitro modelling, CAR-T, checkpoint inhibitors

## Abstract

**Simple Summary:**

The ability of cancer cells to evade the immunological response of the host has been recognized as one of the hallmarks of cancer. Better understanding of this phenomenon is vital for generating more successful anticancer therapies. However, due to the complexity of the tumor microenvironment, classical cell culture techniques have proven insufficient for many aspects of the immune evasion-related research. High hopes for the studies on the tumor immune evasion are currently raised by the recent advances in 3D-bioprinting—a method that allows for precise fabrication of structures containing multiple cell types suspended in biomaterials mimicking the native extracellular matrix. Herein, we discuss the state-of-art 3D-bioprinting techniques and their applications in oncological research, including both existing and potential uses in modeling of immune evasion and response to immunotherapies.

**Abstract:**

In a living organism, cancer cells function in a specific microenvironment, where they exchange numerous physical and biochemical cues with other cells and the surrounding extracellular matrix (ECM). Immune evasion is a clinically relevant phenomenon, in which cancer cells are able to direct this interchange of signals against the immune effector cells and to generate an immunosuppressive environment favoring their own survival. A proper understanding of this phenomenon is substantial for generating more successful anticancer therapies. However, classical cell culture systems are unable to sufficiently recapture the dynamic nature and complexity of the tumor microenvironment (TME) to be of satisfactory use for comprehensive studies on mechanisms of tumor immune evasion. In turn, 3D-bioprinting is a rapidly evolving manufacture technique, in which it is possible to generate finely detailed structures comprised of multiple cell types and biomaterials serving as ECM-analogues. In this review, we focus on currently used 3D-bioprinting techniques, their applications in the TME research, and potential uses of 3D-bioprinting in modeling of tumor immune evasion and response to immunotherapies.

## 1. Introduction

Malignant tumor developing in a living organism can nowadays be seen as a “rouge organ”—a highly complex, internally heterogenous and ever-changing ecosystem [1], that cannot be simply recapitulated by culturing an established cell line in vitro. Moreover, substantial inter-patient variability necessitates a highly personalized therapeutic approach to each of the cancer cases. Therefore, to better understand the tumor biology and to ensure proper testing for the recognized or experimental therapies against individual tumors, one must establish suitable laboratory models.

Although advanced in-vivo-based models, e.g., xenotransplantation of patient-derived tumors to humanized animals [2], may partially reflect the complexity of cancer growth, these studies are tainted with the artificiality of differences between evolutionarily distant species and/or ethical considerations. Therefore, much effort has been employed to generate appropriate in vitro models for reconstituting the interactions within the tumor microenvironment (TME), especially in the context of immune evasion or resistance to modern immunotherapies. Recently, advances in three-dimensional (3D)-bioprinting in vitro methods have raised high hopes for adequate mimicking the interplay between TME and immune effector cells.

## 2. The Role of TME in Tumor Immune Evasion

Initially, cancer is a disease of genes. Hereby, genetic and epigenetic alterations accumulate in cells undergoing malignant transformation, which then alters their behavior and favors the “classical” hallmarks of cancer, as described in 2000 by Hanahah and Weinberg [3]. However, one of the costs of accumulated genetic abnormalities is the generation of new molecular structures, neoantigens, that can be recognized as “non-self” by the immune system [4]. This imposes a selective pressure on the tumor, that, preferably, may lead to the elimination of cancer cells, but can also initiate a Darwinian-like microevolution leading to “survival of the fittest”. In this process, cancer is forced into either dynamic equilibrium with the immune system or, undesirably, into escape from the immune surveillance and into becoming a serious threat to the health and life of an organism. Therefore, immune evasion was added to the list of hallmarks of cancer in 2010 [5]. Although some of the mechanisms of immune evasion can be related to direct interactions between malignant cells and the immune effectors, there are numerous instances when TME serves for cancer as a protector against the immune system and/or immunotherapies [6,7,8].

Depending on a phenotype of a given tumor, TME is a mixture of diverse cellular and acellular components allowing cancer cells to survive, develop and thrive. Within the support of TME for malignant cells, three main paths can be recognized: (1) forming a functional niche for cancer stem cells (CSC) and a scaffolding for proliferating malignant cells, (2) providing oxygen, nutrients, and other soluble factors to sustain the malignant growth, and (3) shielding cancer from the immune system. This last effect can either mimic the natural self-tolerance mechanisms or disable the capabilities of the immune effector cells by non-physiological/exaggerated metabolic pressure. The classical examples of the latter are low pH, hypoxia, oxidative stress, scavenging glucose, and essential amino acids, or even such factors as high extracellular concentration of potassium [9], intrinsic for many tumors.

Regarding the immunosuppressive cellular components of TME, cancer cells tend to selectively attract and reprogram such cells as M2 macrophages, N2 neutrophils, mesenchymal stromal cells (MSC), regulatory T or B cells (Treg or Breg, respectively), or, in some cases, Th17 phenotype of T cells [10]. Each of these cells can be a significant player in the regulatory network hampering the Th1-driven anticancer immune response [11]. At the same time, some of these cells exert innate pro-inflammatory effects that further hamper the specific anticancer immunity [12].

**Figure 1:**
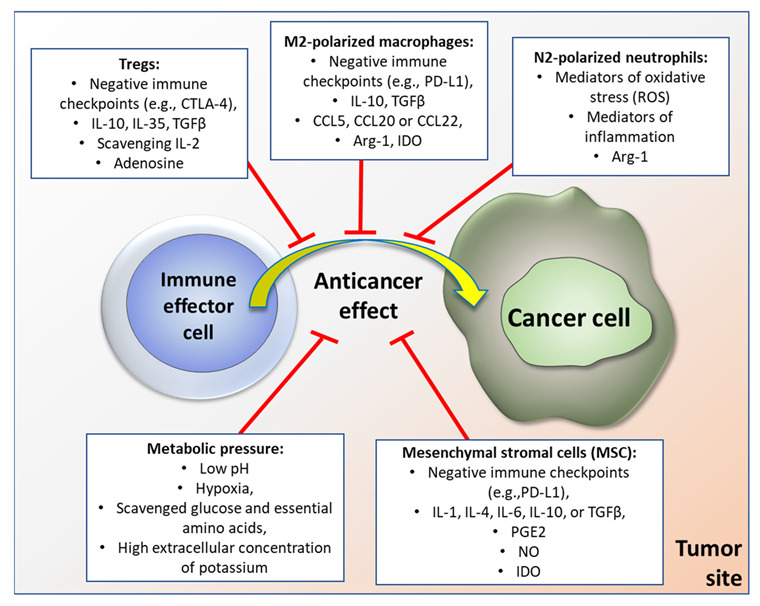
Examples of factors within tumor microenvironment that inhibit anticancer immune response (please see text for details).

For instance, tumor associated macrophages (TAM) of M2 type release immunosuppressive cytokines, such as IL-10 [13], and chemokines (e.g., CCL5, CCL20, or CCL22) attracting Tregs [14]. Additionally, TAM can directly suppress functionality of effector T cells by a range of immune checkpoint molecules, such as PD-L1 or VISTA [15]. Also, TAM express arginase 1 (Arg-1) and generate immunosuppressive metabolites by the indoleamine 2,3-dioxygenase (IDO) pathway, which can induce metabolic starvation in T cells. In turn, tumor associated neutrophils (TAN) of N2 phenotype can, among other effects, sustain inflammation within the TME by producing genotoxic elements such as nitric oxide or mediators of oxidative stress [16]. Robust immunosuppressive and pro-tolerant capabilities are also associated with MSC infiltrates in tumors [17], especially when those cells are induced by such antitumor cytokines as IFN-γ or TNF-α. Similarly to TAM, MSC can also significantly affect specific anticancer response by their metabolic activity (e.g., via the IDO pathway) or production of soluble factors, such as prostaglandin E2 (PGE2), nitric oxide (NO), IL-1, IL-4, IL-6, or TGFβ, and through their direct interactions with other types of immune cell, such as dendritic cells, B or T cells, NK cells, and macrophages [17]. Graphical representation of tumor microenvironment-associated factors hampering the immune response is depicted in Figure 1.

While seemingly random, the spatial architecture of TME is in fact a crucial element for the immune evasion [18]. This notion is strongly supported by recent data collected with state-of-art laboratory techniques, such as deep learning analysis of digital histopathology images [19], probe-based in situ imaging [20], spatial multiomics [21], as well as the advanced biochemistry techniques visualizing gradients of cytokines/chemokines and extracellular nonspecific chemicals (ENSCs) within tumors [18]. Indeed, as immune cells rarely act in isolation, for the successful immune response versus induction of tolerance, an orchestrated cooperation of several cell types is necessary. Therefore, physical intercellular distance, compartmentalization of the tumor mass, and patterns of cellular aggregates within the tumor are of high importance for the immunomodulatory factors that function via cell-to-cell contact or in a juxtacrine/paracrine manner. Another key aspect is the distance from the blood supplying vessels along with the composition and density of extracellular matrix that change the concentration of oxygen and reactive oxygen species, glucose and other nutrients, pH, electrolytes, etc. in a given location. Thus, local conditions for the activity of immune effector cells can be very different within the tumor core, invasive margin, tumor stroma, or early metastatic sites. This can allow survival of residual disease in particular regions of the malignant tumor, even with potentially high effectiveness of the anticancer response or immunotherapy [22]. For a very long time, it was nearly impossible to reconstitute such conditions in vitro for preclinical modeling purposes. This has recently been changing thanks to the spectacular progress in the 3D-bioprinting technology.

## 3. 3D-Bioprinting

### 3.1. Bioprinting Techniques

3D-bioprinting holds one of the greatest promises for the study of cancer biology and the development of novel treatments. Over the past few decades, 2D cancer models have allowed for understanding the fundamentals of cancer biology, but have limited our knowledge on cell-cell and cell-extracellular matrix (ECM) interactions. The ability to use different cell types in combination with biomaterials allows for the precise generation of bioengineered 3D tissues, including representative in vitro tumor models. Three-dimensional cancer models are superior in mirroring cellular behavior and tumor microenvironment, including angiogenesis, metastasis, and anticancer drug resistance.

**Figure 2:**
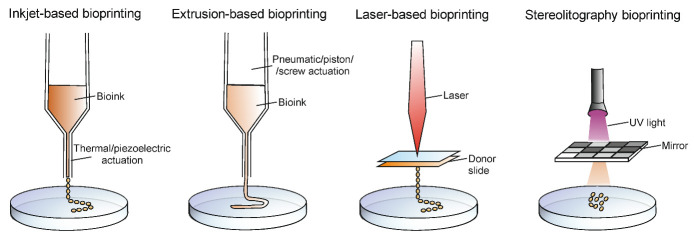
Graphical presentation of principles of four types of 3D-bioprinting techniques.

There are several 3D bioprinting methods that can be distinguished, e.g., inkjet-based bioprinting, extrusion-based bioprinting, laser-based bioprinting, and stereolithography (Figure 2). Every method has unique advantages and limitations, and it is upon the specific requirements of the tissue being produced that the appropriate printing technique is selected [23,24].

Inkjet-based bioprinting is a technique, which involves jetting the ink through the nozzle of a jetting device in the form of droplets, hence it is also called droplet-based bioprinting. This type of bioprinting can be either continuous or drop-on-demand, depending on the mode of deposition of the ink. Among the advantages, fast fabrication speed and moderately high printing resolution (50 μm) can be highlighted. However, bioprinting is available only with low cell densities and is compatible with low viscosity biomaterials (>10 mPa·s). Based on the actuation technique, inkjet bioprinting can be classified into thermal and piezoelectric [25,26,27].

Extrusion-based bioprinting is known as the most popular bioprinting technique. This technique is extensively used, due to the possibility of the use of hydrogels of varying viscosities (30 mPa s to >6 × 10^7^ mPa·s) and bioprinting with high cell densities where cell viability in 3D constructs is very high. However, the limitations are relatively low resolution (>100 μm) and slow printing speed for large structures. Pneumatic or mechanical actuation is used for fluid dispensing, which allows the loaded bioink to be pushed through a nozzle. The advantages of extrusion bioprinting are, undoubtedly, the wide range of printable biomaterials and cost-effective equipment [25,28,29].

Laser-based bioprinting is a less popular technique as compared to the extrusion-based and inkjet-based bioprinting. There are no difficulties with nozzle clogs to overcome as it is a nozzle-free technique. The basis of that technique relies on focusing a laser beam on a donor slide covered with laser-absorbing material, resulting in deposition of small volumes of biomaterials either in the solid or liquid phase, on the target surface. A prominent feature of laser-based bioprinting is a high printing resolution which allows for the manufacture of finely detailed target structures. However, the bioprinting process with laser energy is slower compared to the other methods; furthermore, it is associated with high printing costs, long preparation time, and cellular damage due to ultraviolet curing [25,30,31].

One of the bioprinting methods that allows for production of cellular and acellular structures, while not requiring physical contact, is the 3D-printing with stereolithography (SLA). The applied photocurable bioink is selectively crosslinking into solid hydrogel features by photopolymerization under irradiation. SLA offers high spatial resolution and high cell viability. However, there is a risk of cell toxicity associated with UV usage and cell damage during photocuring [24,25,32].

### 3.2. Bioinks

Bioinks are indispensable components of bioprinting. The term bioink is used to describe a formulation of cells and/or biocompatible materials that serve as scaffolding for living cells in three-dimensional bioprinting. Biomaterials that can be seeded with cells after printing and sterilization are not classified as true bioinks but as biomaterial inks and can be confused with them [33]. Bioinks are generally hydrogel precursors. Thus, during or immediately after bioprinting, the bioink must be cross-linked to give the print the intended model shape [34]. The choice of bioink depends on the specific application, the type of cells used, and the bioprinter. Equally important are the physicochemical and biological properties of the bioink. Properties such as viscosity, gelability, and cross-linking ability significantly influence print fidelity (complexity, resolution, structure size, shape stability) as well as cell viability, proliferation, differentiation, and tissue formation [35]. Bioinks can be classified into two main groups—scaffold-based bioinks and scaffold-free bioinks. The first group is comprised of bioinks where cells are dispersed in biomaterials that mimic the extracellular matrix (ECM), for instance in hydrogels. The second group are the scaffold-free bioinks which contain tissue spheroids, cell pellets, or tissue strands. In this approach, cells aggregate and secrete ECM [36]. Most hydrogel-based bioinks have specific cell-binding sites that are crucial for cell attachment, growth, and differentiation [37]. Among the hydrogel types, one can highlight alginate, gelatin, collagen, fibrinogen, gellan gum, hyaluronic acid (HA), agarose, chitosan, decellularized extracellular matrix (dECM), poly(ethylene glycol) (PEG), and Pluronic [37]. Hydrogels are divided into natural or synthetic groups. The most frequently used natural hydrogels in 3D bioprinting cancer models are alginate, collagen, and gelatin [38]. Alginate is a low-cost biomaterial that features good printability and excellent biocompatibility. However, it shows minimal cell adhesion and slow degradation, but these properties can be improved by the attachment of growth factors or adhesion peptides [39]. One of the notable characteristics of collagen-based bioinks is the possibility to add not only cells but also to enrich their composition with any components of the extracellular matrix [40]. Hydrogels can be combined to improve their mechanical properties, for example, gelatin/alginate/fibrinogen used in glioblastoma [41] or cervical tumor models [42]. Synthetic hydrogels are not as common as the natural ones. However, through chemical modifications, such as functional group cross-linking, as well as domains capable of improving the structural and mechanical properties of bioprinted structures, synthetic polymers can be adapted to the requirements of bioprinting processes [36]. Gelatin methacryloyl (GelMA) hydrogels have been frequently used for various biomedical applications due to their biological properties and tunable physical characteristics [43,44]. The examples of applications of bioinks are presented in Table 1.

## 4. 3D-Bioprinting Applications in Solid Tumor Microenvironment Research

**Figure 3:**
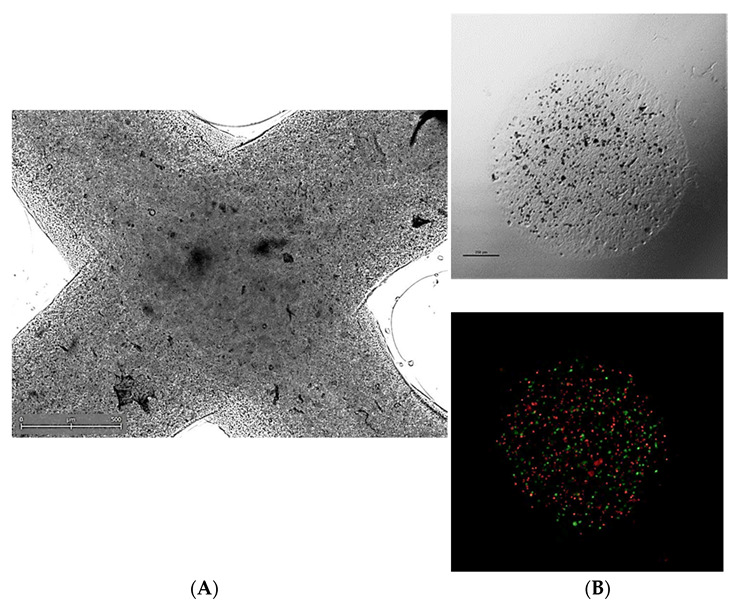
Representative micrographs of 3D-bioprinted co-culture cancer models involving (**A**) extrusion-based method (visible light microscopy) or (**B**) a droplet-based technique in visible light microscopy (upper panel) or fluorescent microscopy (bottom panel)—a mix of PKH67-labeled MSC (green signal) and PKH26-labeled HCT116 colon cancer cells (red signal) was suspended in bioink and co-bioprinted using a BioX bioprinter (CELLINK, Sweden). Courtesy of Ms. Anna Słysz (Maria Sklodowska-Curie National Institute of Oncology, Warsaw, Poland).

In recent years, 3D-printed cell culture systems have gathered attention across various biomedical fields of research. Commonly, pilot studies of novel culture platforms are conducted using established cell lines, usually derived from solid tumors. Optimization of such culture systems for the support of cellular growth, proliferation, and engraftment can be viewed as the beginning of 3D-bioprinted TME research. Example images of such 3D-bioprinted materials are presented in Figure 3.

The level of control over the spatial arrangement of cells, the possibility of multiple cell type use, and fine-tuning the biochemical and physical properties of biomaterials available through bioprinting allows generation of structures of a varied degree of complexity—from simple monocellular models to organoids and assembloids. Organoids are 3-dimensional in vitro constructs mimicking the architecture of specific tissues. Classically, they are derived from induced pluripotent stem cells (iPSCs) or other stem cell types and constructed by cell self-assembly and differentiation under controlled culture conditions [59]. In oncological research, concurrently with the dominant use of tumor stem cells or patient-derived cancer cells (PDCOs), such structures are called tumor organoids or tumoroids. Assembloids exhibit an even greater level of complexity, as multiple cell types are used to recapture the multitude of tissues of which the target organ is built in vivo [60,61]. PDCO-derived organoids are investigated as potential clinical tools for the personalization of oncological treatment and response prediction [62,63,64]. The merger of 3D-bioprinting and organoid technologies both accelerates organoid formation and increases the potential organoid complexity [65]. Many of the structures described later in this section can be considered 3D-bioprinted tumor organoids.

Although 3D-bioprinting has been used in studies focusing on various forms of solid tumors, including neuroblastoma [66,67], melanoma [68,69], pancreatic cancer [55,70], non-small cell lung cancer [71], liver cancer [72], and osteosarcoma [73], the most prolific research has been conducted in breast cancer and glioma/glioblastoma models.

### 4.1. Breast Cancer

TME has been recognized as a crucial element of breast cancer cellular activity, progression, metastasis, and response to treatment [74]. Its effects are modulated by intercellular crosstalk between neoplastic cells and fibroblasts, adipocytes, pericytes, and endothelial cells, as well as through their interactions with the extracellular matrix (ECM) [75]. 3D-bioprinting allows for the creation of artificial culture environments in which it is possible to control the dispersion and the spatial arrangement of specific cell types and furthermore to fine-tune both the biochemical and physical properties (alignment of fibers, porosity, stiffness) of the ECM [76]. Chaji et al. conducted a study in which MCF7 and predifferentiated adipose-derived mesenchymal stem/stromal cell (ADSC)-laden constructs were bioprinted [77]. The cells were found to be viable at day 10 of culture and tended to form clusters within the hydrogel matrix as well as to migrate towards the center of the construct, which the authors interpret as an effect of the hypoxic gradient within the structure. Similar effects were described by Bojin et al., who used a bioink fused with SK-BR-3 cells, peripheral blood mononuclear cells, and fibroblasts isolated from the tumors of breast cancer patients [78]. Through HE staining, they determined that the cells within the construct were able to proliferate and remodel the ECM. The data obtained through the in vitro stage of their experiment was subsequently used to create a virtual model of interactions between breast cancer cells and TME. To assess the potential of 3D-bioprinted models to support the research on paracrine cellular interactions, Horder et al. devised a two-compartment model in which MDA-MB-231 cells were printed onto a hyaluronic acid construct containing adipogenically predifferentiated ADSC spheroids [79]. After 9 days of coculture, the ADSCs within the spheroids presented increased expression of collagen I, VI, and fibronectin, as well as a reduced lipid content compared to the controls. The same cell line was used by Polonio-Alcala et al. in their research of breast cancer stem cell subpopulation [80]. MDA-MB-231 cells were seeded onto a bioprinted polylactic acid scaffold, and subsequently, the potential of the construct to support the expansion of the cancer stem cell niche was confirmed by the increased activity of aldehyde dehydrogenase. An upregulation of stemness marker CD44 was also reported on T47D and MCF7 cell lines cultured on a 2,2,6,6-tetramethylpyperidine-1-oxyl pre-treated cellulose nanofiber bioprinted scaffold [81]. Furthermore, the cells in that experiment have shown an increase in the expression of VIM, a marker of migration. Another study investigated the CD44+ subpopulation of MCF7 cells bioprinted in alginate/gelatin as a platform for drug resistance testing [82]. The authors reported a reduced susceptibility to treatment with camphotectin or paclitaxel compared to unsorted MCF7 cells, confirming the viability of the testing system. Alginate bioprinted scaffolds containing periostin and hydroxyapatite were shown to induce gene expression changes in MCF7 and MDA-MB-231 cells, upregulating the marker genes for stemness (*CD44*, *ETV1*, *MALAT1*), epithelial-mesenchymal transition (*TWIST1*, *SNAI1*, *MUC1*), and downregulating the markers of cell proliferation (*MKI67*, *CCNA2*) and differentiation (EPCAM) relative to 2D culture [83]. Additionally, the transcriptomic pattern of response to doxorubicin and 5-fluorouracil differed between the cells cultured in 3D versus 2D. Han et al. focused on metastatic disease, specifically estrogen-receptor-positive breast cancer metastases to bone tissue [84]. First, they fabricated an osteomimetic DS-3000 resin scaffold using stereolitography, which was subsequently seeded with patient-derived cells isolated from bone lesion biopsies. Through live/dead staining and an altered expression of Ki67 and EpCAM, the authors concluded that the scaffolds supported cell proliferation. Scaffolds of the same type were also seeded with patient-derived triple-negative breast cancer cells and were found to promote cisplatin resistance in relation to 2D culture. Cui et al. have devised a three-compartment model to study breast cancer bone invasion [85]. Their model consisted of 3D-bioprinted compartments of bone (nano hydroxyapatite-laden gelatin [GelMA] and polyethylene glycol diacrylate [PEGDA] ink), blood vessel (GelMA), and tumor (GelMA/PEGDA) seeded with osteoblasts, human umbilical vein endothelial cells (HUVEC), and MCF7 or MDA-MB-231 cells, respectively. The constructs supported cell proliferation and migration, especially of the latter, invasive cell line. Mollica et al. used an entirely different approach to ECM preparation, as they developed a self-gelating hydrogel created from decellularized rat or human breast tissue [86]. MCF-7 and MDA-MB-468 cells were printed onto the scaffolds and cultured for up to 14 days. Subsequent analyses have shown that the constructs promoted cell engraftment and invasion and supported organoid/tumoroid formation.

### 4.2. Glioma

The TME of gliomas, especially that of glioblastoma multiforme (GBM), can be divided into three main components: the ECM, the surrounding cells, and the hypoxia gradient [87]. Wang et al. used glioma stem cells of the GSC23 line, suspended them in an alginate/transglutaminase/fibrinogen/thrombin bioink, and subsequently printed a 3D grid-like structure [88]. The cells within the construct displayed stable proliferation and in time formed spheroids, which were harvested for analyses at day 15. The authors reported an increased concentration of VEGF in the culture medium relative to 2D controls, as well as an increased expression of CD31, VEGFR2, HIF-1α, and CD133 compared to suspension-cultured controls, which was interpreted as a stronger angiogenic potential. In another study, the team focused on U118 cells, and using the same printing method, investigated the markers of epithelial-mesenchymal transition (EMT) [89]. Compared to 2D controls, 3D-cultured U118 cells demonstrated an increased transcriptional activity of genes encoding molecules related to EMT: HIF-1α, Snail, Twist, and VEGF. In a subsequent study, GSC23 and U118 cells were suspended in alginate, then bioprinted and cultured to form spheroids [90]. The cells of both types were resuspended and implanted into nude mice. GSC23 cells presented a greater capacity to transdifferentiate into an endothelial-like phenotype and formed lumen-containing structures within the tumor. In another study by the same team [47], bioprinted fibers comprised of a GSC23 shell and U118 core to mimic the pathophysiological cellular arrangement in vivo. These constructs exhibited an increased expression of markers of tumor invasion and drug resistance (matrix metalloproteinase-2 and -9 [MMP2, MMP9], vascular endothelial growth factor receptor-2 [VEGFR2], and O6-methylguanine-DNA methyltransferase [MGMT]) when compared to solely U118-printed fibers. Hermida et al. used alginate/RGD (Arg-Gly-Asp peptide sequence) bioink, which they first tested with U87MG glioblastoma cells and MM6 monocytes/macrophages [91]. After proving the viability of the model, their main experiment focused on glioblastoma stem cells (G7/G144/G166 cell lines) coprinted alongside glioma associated stromal cells and microglia. Compared with 2D cultures, bioprinted glioblastoma stem cells did not lose the expression of the pluripotency marker Nestin or gain the expression of the glial lineage marker GFAP, even after ceasing their exposure to EGF and FGF in the culture media. Bioprinted U87MG ang G7 cells demonstrated greater resistance to temozolomide and cisplatin compared to traditional culture. The addition of MM6 to the U87MG-containing bioink further increased the resistance to cisplatin. Another team studied the effects of ECM stiffness using GMHA (stiff) and GelMA (soft) bioinks [92]. The stiff culture conditions altered the transcriptional profiles of TS576 cells, elevating the expression of *CHI3L1*, *IFIT1*, *OAS1*, *TMEM45A*, *SAMD9*, *IFI6*, *NDRG1*, *FN1*, *AQP4*, *AL136131.3*, *SPP1*, *APOL4*, *VEGFA*, *SCG3*, *APOL6*, *DDX58*, *CA IX*, *HIF1A*, *SLC2A1*, and *PROM1*—genes associated with hypoxia-induced chemoresistance, adhesion, proliferation, angiogenesis, tumor edema, migration, and immune evasion of glioblastoma. The incorporation of HUVEC cells to the stiff model produced sprouted blood-vessel like protrusions in close contact with the SOX2+ TS576 cells. Both 3D culture conditions increased the TS576 resistance to temozolomide, although the effect was stronger in the stiffer ECM, and further amplified by the addition of HUVECs. Neufeld et al. fabricated a complex perfusable model of glioblastoma using a fibrin bioink containing patient-derived glioblastoma cells, human astrocytes and human microglia [93]. The vessels of the construct were sacrificially printed and seeded with HUVECs and human microvascular brain pericytes. Fibrin bioink was subsequently used with the cells’ murine counterparts, and the transcripromic profiles of 2D-, 3D-printed, and murine in vivo models were compared, showing close similarities in gene expression of cells cultured in vivo and in the 3D bioprinted system. Heinrich et al. used a GelMA/gelatin bioink to study the interactions between murine GL261 glioblastoma cells and murine RAW264.7 macrophages in a sophisticated model of 3D-bioprinted mini-brain [53]. Compared to solely paracrine mediated contact conditions, RAW264.7 in the co-printed model exhibited increased expression of *Fgf2*, *Il-1β*, *Arg-1*, *Nos2*, *Fgfr1*, *Il-10*, *Il-6*, *Mmp2*, and *Mmp9*, which are consistent with the transcriptional profile of glioblastoma associated macrophages. In turn, the GL261 cells of the co-printed model shown increased expression of *Spp1*, *Gfap*, *Chil1*, *Olig2*, *Pdgfrβ*, and *Timp1* alongside genes responsible for EMT—*Vim*, *Nest*, and loss of E-cadherin expression.

## 5. 3D-Bioprinting Applications in Hematological Neoplasm Microenvironment Research

Hematological malignancies, often called non-solid cancers, are a heterogenous group of neoplasms usually arising from the lymphoid or myeloid lineage. The most typical niches of these cancers include the bone marrow (leukemias, multiple myeloma, myeloproliferative neoplasms) and lymphoid tissues (lymphomas). In vitro modeling of these diseases remains a challenge due to the structural complexity and dynamic nature of the TME (multiple cell types, myriad of para- and juxtacrine interactions) [94]. Thus, there is a scarcity of published research involving 3D-bioprinted constructs. Nevertheless, Sbrana et al. produced a long-term 3D-printed chronic lymphocytic leukemia (CLL) model using patient-derived CLL cells and MEC1 cell line suspended in bioink composed of third-party hydrogels (CELLINK) [95]. They have observed a superior viability of the cells in 3D environments (cultured up to 28 days) and significant differences in gene expression between 3D- and 2D-cultured MEC1 cells. They report upregulation of *CXCR3*, *CCL22*, *SELL*, *HCLS1*, *AICDA* (genes associated with leukocyte trafficking, integrin activation, cytoskeletal remodeling, adherence of lymphocytes to endothelial cells in peripheral lymph nodes, migration, trafficking and homing of CLL cells, somatic hypermutation, gene conversion, and class switch recombination in B-lymphocytes), and downregulation of protooncogenes MYC and PIM3. Patient-derived CLL cells sustained their CD19 and CD5 phenotype for the entire length of culture, as well as an increased surface IgM expression. Compared to the 2D culture, 3D-printed primary CLL cells exhibited a decreased expression of proapoptotic BAX and an increased expression of antiapoptotic BCL2. Braham et al. devised a multiple myeloma model in which patient derived CD138+ cells were co-cultured alongside MSC and endothelial progenitor cells (EPCs) on a bioprinted calcium phosphate cement scaffold [96]. CD138+ cells were viable up to 28 days and sustained proliferation, reaching an average relative contribution to the construct of 40.8% on day 28, from 6.8% on day 7. Wu et al. co-cultured multiple myeloma cells with HS5 stromal cells in a bioprinted construct (stiff mineral outer layer, soft hydrogel core) and compared cell viability to 2D culture [97]. The constructs supported cell viability for 7 days, whereas the 2D environment for only up to 5 days.

Post-transplantation lymphoproliferative disease (PTLD) is a peculiar form of lymphoid neoplasm as it only arises in the immunosuppressed population and is characterized by relatively frequent extranodal involvement when compared to lymphomas arising in immunocompetent patients [98,99]. According to the current WHO classification of lymphoid neoplasms [100], the monomorphic subtype includes all currently recognized types of non-indolent lymphoid neoplasms (including multiple myeloma). These facts combined, there seems to exist a possibility to circumnavigate the challenges of replicating the bone marrow and lymphatic tissue niches by using PTLD cells already adapted for extranodal growth. A successful non-bioprinted 3D spheroid model of imunocompetent DLBCL co-cultured with ADSCs devised by Foxall et al. [101] confirms the relevance of aggressive lymphoma culture in solid environments. Potential PTLD models would serve only as an approximation of lymphomas in the immunocompetent, as the mechanisms driving the development of malignancy are different from their immunocompetent counterparts—PTLD’s development is more frequently associated with the Epstein–Barr virus (EBV) infection, decreased immune surveillance due to immunosuppression, chronic inflammation, and immune exhaustion [102,103].

## 6. 3D-Bioprinting in Modeling Tumor Evasion Mechanisms

As tumor immune evasion mechanisms are recognized to be a key component in the development, propagation, and treatment resistance in malignancy, it is of utmost importance to create in vitro the immune-competent tissue models for studying the tumor immunobiology and evaluation of immunotherapies. Due to the high complexity of this task and the emerging nature of 3D-bioprinting techniques, this research is at very early stages of development. Yet, the initial studies are extremely promising. At least several models of evasion have already been generated (reviewed in [104]), focusing on,. e.g., hiding from detection by the immune system (loss of MHC-I and -II, CD58, CD86, CD80, CD54), resistance to apoptosis (expression of PI9, cFLIP, overexpression of BCL2, loss of FAS or TRAIL), direct inhibition of immune cells (expression of PD-L1 and 2, HLA-G, FASL, CD47), and creation of an immunosuppressive TME (secretion of IL10 and TGFβ, recruitment and promotion of Treg and TAM). As described earlier in the text, a number of 3D-printed models exhibit molecular signatures of supporting cell dedifferentiation, which is associated with the expression of CD80, PD-L1 and NKGD2 [105]. Examples of other attempts to study the immune evasion with 3D-bioprinting are presented below.

Kim et al. fabricated a bioprinted bladder cancer model using T24 and 5637 bladder cancer cell lines alongside HUVEC and MRC-5 human lung fibroblasts to evaluate its effect on THP-1 human monocyte response to Bacillus Calmette–Guerin (BCG) [106]. They report a favorable secretion profile of THP-1 (IL6, IL12, IFN-γ and TNF-α), concurrent with clinically observed effects of BCG treatment.

Chirivi et al. used poly(ethylene glycol)-fibrinogen (PEG-FB) hydrogels of varying stiffness mimicking those of ‘healthy’ ECM and that of triple-negative breast cancer on the phenotype and gene expression of infiltrating CD4+ and CD8+ lymphocytes from healthy donors [107]. They reported a decrease in cell volume in both resting phase T-cell types, more pronounced in the stiffer matrix. The effect was opposite in activated T-cells—both exhibited a marked increase in cell volume. Independently of their phase, both cell types exhibited an increase of nuclear diameter in the stiffer matrix. Furthermore, the transcriptome of activated CD8+ cells in the stiff matrix has shown a downregulation of NFKB1, NFKB2, RelA, and SP1, which coincided with a decrease of IFN-γ production—which the authors claim to represent a decreased cytotoxic potential. Activated CD4+ cells upregulated NFKB1, RelA, and A20, which is conducive to Treg phenotype development.

Although not specifically in bioprinted systems, various properties of 3D culture environments have been shown to affect immune cell function. ECM stiffness, topography, pressure gradient, and fiber organization influence immune cell mobility [108,109,110]. Hypoxia gradient of the tumor and associated acidification of the TEM along with high lactate concentrations hamper NK and T-cell cytolytic activity, proliferation, and cytokine production [111]. These factors are easily controlled in 3D-bioprinting systems. Melanoma cells cultured in 3D environments have been shown to produce greater concentrations of IL10 than 2D controls, allowing for the impairment of monocyte-to-dendritic-cell differentiation and subsequent M2 macrophage emergence [112].

Regarding immunotherapies, a notable work was done by Grunewald et al. [113]. Specifically, this research team produced a 3D-bioprinted neuroblastoma model for the optimization of chimeric antigen receptor T-cell therapy (CAR-T) [113]. Through immunofluorescent microscopy, it was possible to confirm CAR-T’s ability to infiltrate the bioprinted construct and lyse an average of 72% of neuroblastoma cells post-exposure. By proxy, this also proves the rationale of 3D-bioprinted model usage as platforms for studying the interactions between the immune system, malignant cells, and their TME, and also testing anticancer immunotherapeutic approaches, such as other adoptive therapies [114] or immune checkpoint-modulating agents [115].

In summary, it is becoming obvious that 3D-bioprining methods are highly promising in studies on tumor immune evasion. As several models have already been established, one should expect further development of these approaches in tumor immunobiology research. However, some obstacles are evident, such as difficulties in mimicking blood flow or lymphatic efflux, and thus mimicking the metastatic process. Perhaps combination of 3D-bioprinting with microfluidics could be a solution for generating biomimetic tissues for tumor-related studies [116].

## 7. Conclusions

In this review, we discuss the recent advances in 3D-bioprinting methods from the perspective of preclinical modeling of intrinsic molecular mechanisms within TME that can induce T cell dysfunction and hamper the anticancer immune response. It is envisioned that due to a new generation of increasingly sophisticated 3D in vitro models, we can progressively better understand these underlying mechanisms and lay a foundation for new successful immunotherapies that would battle this deadly disease.

## Figures and Tables

**Table 1:** Examples of applications of bioinks in 3D-bioprinting models of cancers.

Bioink	Bioprinting Technique	Biochemical/ Physical Properties	Crosslinking Mechanism	Applications
	Naturally-derived hydrogels			
Collagen	EBB ^1^	The major component of ECM in most tissues, cell-friendly, possesses natural cell binding sites, hydrophilic, well-studied temperature-dependent gelation, low immunogenicity	pH, thermal	Glioblastoma-on-a chip [45]; Breast tumor model [46]
Alginate	EBB DBB ^2^	Easy and quick cell encapsulation, low cell attachment, high porosity, hydrophilic	CaCl_2_, CaSO₄	Glioblastoma-on-a chip [47,48]; Melanoma model [49]
	Synthetically-derived hydrogels			
Pluronic F127	EBB	Low toxicity, reverse thermal gelation, high drug loading capabilities, ability to gel in physiological conditions at relatively low concentrations, biologically inert towards multiple cell types, broad range of viscosities	thermal	Hepatocarcinoma model [50]
Polyethylene glycol (PEG)	LBB ^3^ (streolitography)	Hydrophilic, enhanced biocompatibility, resistant to protein adsorption and cell adhesion, nonbiodegradable, poor mechanical strength	photocrosslinking	Breast tumor model [51]
GelMA	LBB, LBB (streolitography) EBB	Good solubility, low antigenicity, combined biocompatibility, has bio-active peptide sequences, mimics native ECM	photocrosslinking	Hepatocarcinoma model [50]; Ovarian cancer [52]; Glioblastoma-on-a chip [53]; Invasive ductal carcinoma model [54]; Exocrine pancreas spheroid model [55]
	Combined hydrogels			
Gelatin, alginate and fibrinogen	EBB	Combined components of extracellular matrix that is bioprinting-friendly	thrombin, CaCl_2_, thermal	Glioblastoma-on-a chip [41]; Cervical tumor model [42]
Alginate and gelatin	EBB	The combination of these two substances provides a substrate with enhanced mechanical and structural properties	CaCl_2_	Breast tumor model [56,57]; Lung cancer model
Methacrylated hyaluronic acid and gelatin	EBB	Increased final mechanical strength by methacrylation	photocrosslinking	Breast tumor model [58]

^1^ EBB—Extrusion-based bioprinting. ^2^ DBB—Droplet-based bioprinting. ^3^ LBB—Laser-based bioprinting.

## Data Availability

Not applicable.
